# Development and implementation of the IMRA multiplatform Foundations in Robotic Surgery online learning curriculum

**DOI:** 10.1007/s11701-025-03091-w

**Published:** 2026-02-26

**Authors:** Tayla Fay, Daniel Costello, Dean Driscoll, Niall M. Corcoran, Anthony J. Costello, Henry Woo, Helen Mohan

**Affiliations:** 1 The International Medical Robotics Academy, Melbourne, VIC Australia; 2 Department of Surgery, The University of Melbourne, Melbourne, VIC Australia; 3 Department of Urology, Western Health, Melbourne, VIC Australia; 4 Department of Urology, Blacktown Hospital, Blacktown, NSW Australia; 5 Blacktown Mount Druitt Clinical School, Blacktown Hospital, Blacktown, NSW Australia; 6 Department of Surgery, Peter MacCallum Cancer Centre, Melbourne, VIC Australia

**Keywords:** Robotic surgery, Online, Curriculum, Distance education, E-learning

## Abstract

A major limitation to robotic training internationally is sufficient access to console and bedside operating time. It is important that trainees are “robot ready” when clinical opportunities arise. Delivering the didactic component of training in advance of hands-on training opportunities is needed to maximise the efficiency of console time. This study describes the development and implementation of a platform agnostic online curriculum (Foundations in Robotic Surgery -IMRA) that provides an overview of robotic surgery as didactic learning prior to hands-on experience. Curriculum development is reported using Kern’s model. A literature review was conducted in accordance with the PRISMA statement. A mixed-methods approach, incorporating surveys and semi-structured interviews, defined learning objectives and curriculum content. Curriculum outcomes were assessed via surgeon surveys, and modifications were made based on feedback from the Royal Australasian College of Surgeons. The literature review revealed a gap in vendor-neutral online curricula for robotic surgery. Six experts comprising of general surgeons and urologists, identified key learning objectives on safety, communication, and surgical technique. This gap was addressed by developing an online curriculum comprising of ten theory modules. In the development group (*n* = 28; 16 responses), Kirkpatrick assessment showed a 60% increase in participants rating their knowledge as good or excellent. Confidence in bedside assistance and console training rose by 37.5%. In the rollout group (*n* = 108; 88 responses), 89.8% rated the content quality as good or excellent. Development and implementation of the IMRA Foundations in Robotic Surgery Curriculum demonstrated improvements in learners’ self-rated knowledge and confidence in both bedside and console aspects of multi-platform robotic surgery.

**Supplementary Information:**

The online version contains supplementary material available at 10.1007/s11701-025-03091-w.

## Introduction

Access to robotic surgical training is limited internationally, despite a rapidly expanding need for robotically trained surgeons. It is crucial to establish and implement valid and comprehensive training programs to prioritise patient safety and improve patient outcomes [1–5]. Given the time-pressured environment of most robotic hands-on training, there is a demand to move the didactic component of training into online self-paced learning.

Online learning has gained popularity in recent years, accelerated by the COVID-19 pandemic, due to its convenience, flexibility, and scalability, overcoming geographical boundaries and time constraints [6–9]. Online learning resources can employ a combination of written text and multimedia elements [3]. An advantage of an online learning approach is the opportunity to provide standardized and structured learning experiences, that can be adapted to an individual’s learning needs, and facilitate continuous learning and skill development [11]. There are many studies showing effective implementation of online learning, e.g. Kerfoot et al. demonstrated a statistically significant knowledge gain from case-based teaching in urology delivered through online learning [12]. Asynchronous delivery allows surgeons to learn at their own pace without disrupting their clinical commitments. Moreover, online modules can be customized to cater to the unique requirements of each specific surgical procedure and may incorporate assessments and individualised feedback to enhance learning [13].

Kern’s has provided a useful framework for considering curriculum development (Fig. 3). The six steps in Kern’s method span from learning needs assessment to evaluation and feedback of the course itself. The Kern’s method is a well-accepted educational pedagogy. IMRA has developed a comprehensive curriculum for robotic training using this principle and spanning from basic generic skills to specialty specific skills. This initial online course was designed as a prelude to all the rest of the educational content offered by IMRA.

This report delineates the development and initial implementation of the IMRA Foundations in Robotic Surgery (FORS) curriculum. The curriculum encompasses basic principles of robotic surgery and aims to equip trainees with the skills to maximise efficiency of hands-on robotic training opportunities, including the next step in the IMRA curriculum pathway which is progression to VR simulation and then hydrogel (Pindari™) based robotic simulation.

This study aimed to describe the development and implementation of the IMRA Foundations in Robotic Surgery online, video-based, educational curriculum. Outcomes were assessed using the Kirkpatrick framework.

## Methodology

### Curriculum design

To prepare participants for the hands-on robotic simulation training an online foundational course teaching the theory and principles for safe robotic surgery was created. Kern’s six step approach to curriculum development for medical education was used for course design [14, 15].

Step 1: Problem Identification and General Needs Assessment

A scoping review was conducted in accordance with the PRISMA-SCr guidelines [16]. A comprehensive search was conducted in PubMed, Embase, and Scopus databases, supplemented by manual searches of the reference lists. (Appendix A.) The eligibility criteria included studies on online learning resources in robotic surgery education, published in English from 2010 to 2023, with a focus on e-learning courses or curricula containing e-learning modules.

**Fig. 1:**
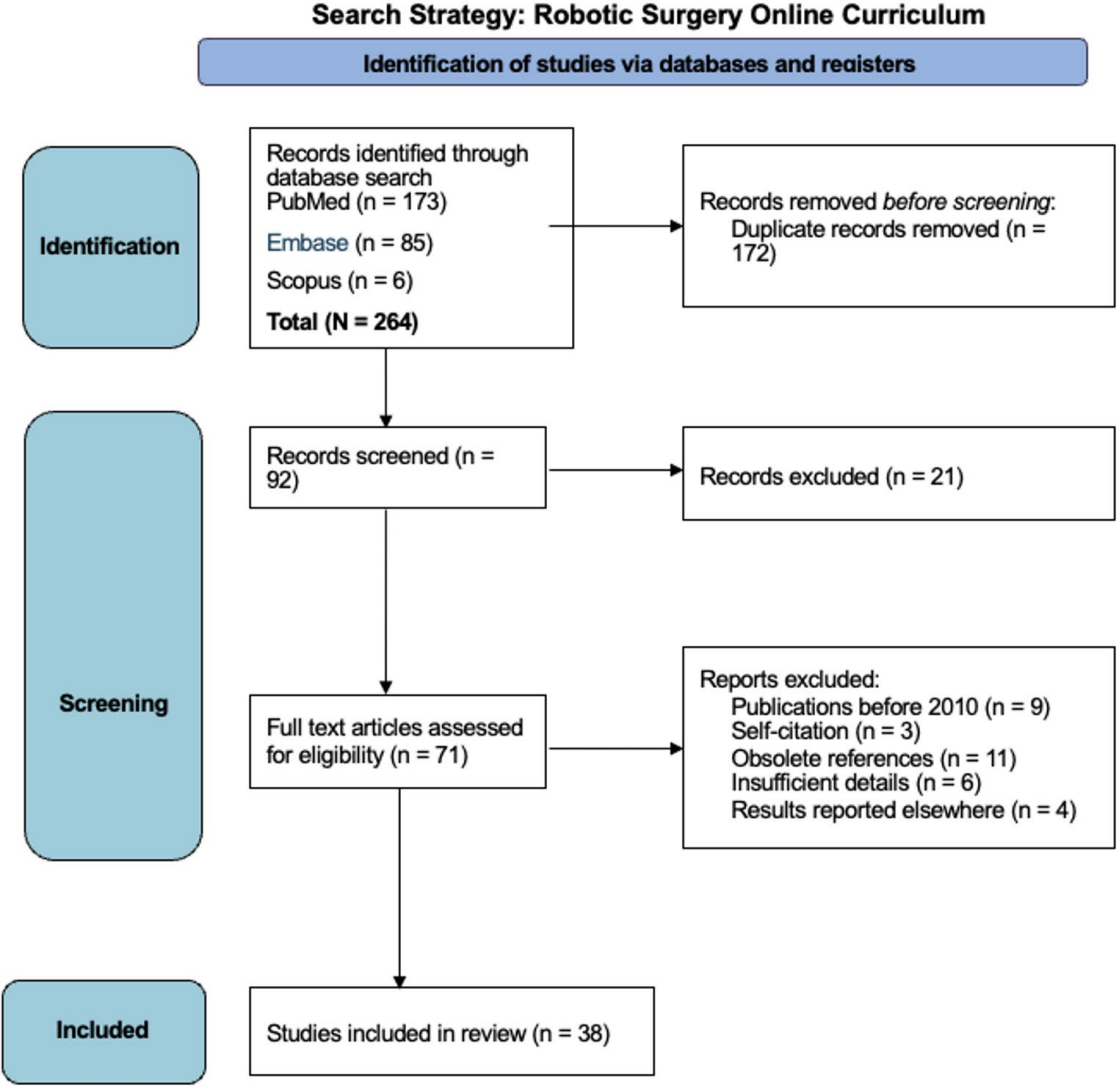
Search Strategy

In addition, grey literature searches were conducted using the Google search engine, through a Chrome anonymous browser to ensure a de-personalized search result. A further grey literature search included textbooks on robotic surgery Fig. 1.

Step 2: Targeted Needs Assessment

A targeted needs assessment was conducted using an online consensus group of experts and a review of existing robotic surgery textbooks [1, 17–19]. Six robotic surgery experts who had experience with directing robotic surgery fellowship programs agreed on key learning needs and agreed upon 10 key theory modules. (Modules 1–10 in Table 1). These robotic surgery experts were from Australia, the United Kingdom and the United States of America. A senior learning designer was engaged to facilitate the development of learning objectives and assessments.

**Table 1:** Foundations of robotic surgery online course outline

Module	Title	Content
1	Orientation	Overview of the curriculum, simulation technology and surgical robots used in robotic surgery education.
2	Introduction	History of robotic surgery and emerging robotic surgical systems.
3	Robot setup	Robot system features, instructional videos on port handling, docking and instrument insertion.
4	Anaesthetics for robotic surgery	Perioperative and anaesthetic considerations for robotic surgery.
5	Console setup	Instructional videos on ergonomics and instrument handling.
6	Fundamentals of operating	Basic robotic surgical skills.
7	Assisting in robotic surgery	Bedside training for the robotic surgical assistant.
8	Achieving robotic surgical competency	Lectures from simulation training experts.
9	Non-technical skills for robotics	Lessons from the aviation industry for robotic surgeons.
10	Vendor training	Preparation for driving the da Vinci Xi robot.

Step 3: Goals and Objectives

The goals and objectives were initially determined by the experts in the field and subsequently refined after the first round based on feedback from surgical consultants and registrars. The interviews conducted aimed to understand their specific educational requirements and experiences with the course.

Step 4: Educational Strategies

An online curriculum was developed comprising of 10 modules initially. The 11th module ‘Preparation for Basic Surgical Training Course’ was added after evaluation and feedback was provided in step six (Table 1). All modules were compulsory and designed to be completed sequentially. The total course length was approximately 10–15 h.

The curriculum was designed to provide flexible and remote access to high-quality education, designed for trainee robotic surgeons, and delivered by experts in the field. Learners could complete the course at their own pace and re-visit the modules as required. The Kirkpatrick framework was employed to evaluate the success of the program. Knowledge checks were designed to engage adult learners and solidify key points in each module.

To evaluate the success of the curriculum delivery an online assessment was administered after every module. The assessments measured several factors, including topic length, content quality, usefulness and relevance, as well as the knowledge of the presenter and the quality of the presentation and course materials.

### Implementation, evaluation and feedback - steps 5 and 6

The curriculum was implemented and underwent three rounds of evaluation and feedback. The first round of feedback was provided by the participants who completed the version of the course. (15 consultant surgeons and 13 trainees). For the second round of feedback the Royal Australasian College of Surgeons (RACS) provided a panel of reviewers.

## Results

### Literature review

In total, 264 relevant articles were identified, and after screening the titles and abstracts, a total of 38 full-text original articles were included in the literature review. The majority of studies were conducted in the United States and Europe, with focus on urology, thoracic surgery, gynaecology, otolaryngology and general surgery.

Four robotic surgery textbooks were included in the grey literature search [1, 17–19]. They generally included; an introduction to the robotic surgery machine, the history of robotic surgery, advantages and limitations of robotics, basic bedside setup, basic console skills, troubleshooting and procedure-specific considerations. Some textbooks included a training and credentialling section and anaesthetic considerations. Non-technical skills were not covered, or only received a cursory mention.

### Themes identified in existing online resources

The content of existing online resources covered mainly technical aspects of robotic surgery: Docking and undocking, safe robotic surgery setup, aligning the robotic arms and connecting the instruments. Safely undocking the robot including detaching the instruments, safely transporting it, patient positioning, port placement, surgeon ergonomics. Console skills including optimising visual control during robotic surgery, instruments and basic controls [20–35]. 

The structure of online robotic surgery teaching resources varied- most resources included a combination of didactic lectures and instructional videos, with some including case presentations, and interactive discussions.

Similarly, textbooks generally included an introduction to the robotic surgery machine, the history of robotic surgery, advantages and limitations of robotics, basic bedside setup, basic console skills, troubleshooting and procedure specific considerations. Some textbooks included a training and credentialling section and anaesthetic considerations.

### Targeted needs assessment

Six robotic surgery experts who had experience with directing robotic surgery fellowship programs considered the themes covered in the existing resources and textbooks and outlined the 10 key theory modules for an online robotic surgery course. These robotic surgery experts were from Australia, the United Kingdom and the United States of America. Non-technical skills were minimally covered or not covered at all in current resources, and this was identified as a key gap in the literature that the Foundations in Robotic surgery aimed to address.

### Course development

#### Course structure

An online format was selected to be pragmatic for participants. This course was designed during the COVID-19 pandemic in Australia, where face-to-face surgical education was suspended for almost 2 years, highlighting the importance of remote surgical learning. In the initial development and testing group (*n* = 28, 24 responses) 45.8% reported a decrease in their surgical caseload during 2020, varying from a minimal decrease to no operating for four months out of the year. Participants reported a median of 75% of their professional development to be online. Notably, 23.1% reported 100% of their professional development occurred online. The proportion of online professional development in 2020 (mean **=** 58.1**%**) was notably higher than in 2019 (13.8%).

This has relevance beyond covid as the majority of target learners were typically time-poor surgeons or surgical trainees and online delivery remains a pragmatic way of delivering didactic content.

**Fig. 2:**
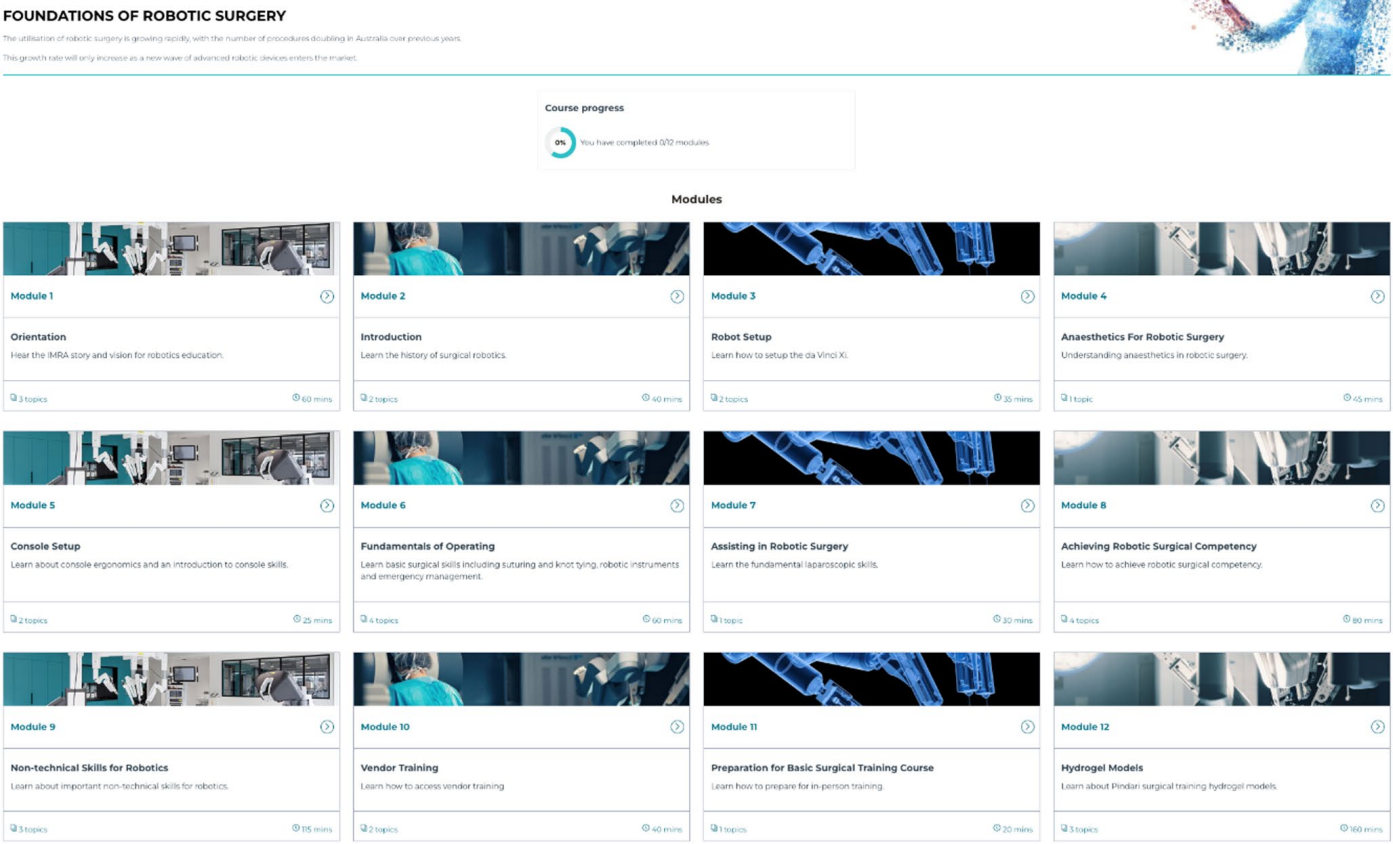
Info-graphical outline of the IMRA Foundations course modules

The overall goal was to ensure learners were safe and informed when entering the next phase of simulation and dry lab exercises in their robotic training. Structured interviews were conducted with 6 registrars and 1 consultant surgeon who had completed the Foundations Course, in order to gain detailed insights about their educational needs and experiences of the course. The objective was to ensure that learners had a solid grasp of the theory and principles of robotic surgery before embarking on hands-on training in hospital or laboratory settings, thereby optimizing the efficiency of face-to-face learning encounters. Short videos and imagery-based language were employed to craft engaging and informative content Fig. 2.

The following specific objectives were set based on these interviews: Know the steps for safe robot setup, be aware of anaesthetic safety considerations for robotic surgery, know the principles of good robotic surgical techniques at the console and at the bedside, understand how to achieve robotic surgical competencies and recognise the importance of human factors in robotic surgical practice.

### Course implementation and evaluation

The course was initially rolled out as a beta-testing phase. Following evaluation and feedback, changes and modifications were made to ensure it met learners’ needs. External review of the course was carried out by the Royal Australasian College of Surgeons and RCSI, and modifications made based on feedback.

### Initial testing phase

Twenty-eight participants (15 consultant surgeons, 13 residents/registrars) were invited by email to complete this online course. Fifteen qualified robotic surgeons (12 male, 3 female) with a median robotic experience of 150 cases, completed version 1, the initial development and testing of the online course. Post-course structured interviews and surveys regarding the course quality and content were conducted with these robotic surgeons (see step 6). Thirteen surgical and gynaecology trainees (7 male, 6 female) from Australia and New Zealand completed the foundational course in an average of eight hours. None of these trainees had performed robotic surgery. Twelve trainees had observed or assisted robotic surgery. One trainee had neither observed nor assisted with robotic surgery.

A gap was identified when participants were surveyed about their current access to robotic surgery training prior to the course, 53% reported dissatisfaction.

Before completing the course, 6 participants rated their knowledge as poor, 5 as fair, 2 as good, and none as excellent. After the course, only 1 participant rated their knowledge as poor, while 6 rated it as fair, 4 as good, and 1 as excellent, 1 no response.

Confidence levels in bedside assisting also improved post course.

Similarly, participants initially rated their confidence in undertaking robotic console training as (7) poor, (4) fair, (2) good. Following the course, only (1) participant rated their confidence as poor, while (4) rated it as fair, (5) as good, and (2) as excellent, (1) no response. This indicates improved confidence levels in undertaking robotic console training after the course. Upon completion of the course 62.5% of participants agreed they would recommend the course to a colleague.

One participant remarked; “I think this course and the practical elements should be mandatory for SET urology trainees and it should really be part of our curriculum and funded by the college from our fees. It is the future of minimally invasive surgery and our specialty is on the forefront of it.”

Three major rounds of evaluation and feedback were completed. The initial round was completed by the participants who undertook the initial development and testing phase of the course (*N* = 28). Thirteen (11, urology trainees and 2 OBGYN trainees) and 15 consultants ( eight urologists, four OBGYN and three colorectal surgeons) The course predominantly received positive feedback across all domains including; content relevance, quality and usefulness as well as presenter expertise, presentation and course materials quality. (Table 2.) Some participants suggested shortening the course length and being more concise in some lectures. Additionally, a few minor issues were noted, such as typos in slides and unconventional file names for PowerPoint attachments.

**Table 2:** Survey responses for “rate the attributes of the foundations course as a whole”

	Excellent	Good	Fair	Poor
Topic length	3	13	0	0
Content quality	8	8	0	0
Content relevance	10	5	1	0
Calibre of presenters	12	4	0	0
Presentation quality	9	6	1	0
Course materials quality	5	10	1	0
Online format & interface	12	4	0	0

The learning designer reviewed the scores and comments for each individual topic to identify those rated as poor or fair. These topics were then reviewed by the content experts and then redeveloped and improved, according to these scores and comments. This included recapturing lectures, videos and changing supporting text and content. An 11th module was added, with the aim of preparing learners for VR and hydrogel based simulation exercises.

The second round of evaluation was completed by the Royal Australasian College of Surgeons. The design and production of the course was rated very highly and required no changes. Changes to the format of the content were recommended. This included allowing students greater autonomy to complete the modules in any sequence that they chose, as opposed to dictating a linear approach. This was suggested to allow greater flexibility for students to complete the course.

Additional literature material and evidence of validity were added to explain and support the educational pathway. Text summaries and review questions were added to topics to foster deeper engagement with the video content in the course. More detailed learning outcomes were added to the topics to clarify the important aspects of the course content. The reviewers questioned the need for all modules to be compulsory and suggested that some modules could be made flexible for “just-in-time learning” to accommodate time-poor surgeons. Moreover, they suggested learning outcomes for each individual module should be included as well as a lesson structure with explicit information on what will be learned, why it is important, how it will be learned, the learning activity, knowledge check, and closure.

During the implementation and rollout phase, 108 participants completed the course, with 60 completing both the pre- and post-course surveys. The quality of course content was rated as good or excellent by 90% of participants. The relevance of the content was rated good or excellent by 81%. The presenters’ knowledge was similarly rated as good or excellent 98%. 35 participants gave feedback on the most valuable aspects of the course as free text feedback, 37% identified the non-technical skills module to be one of the most valuable modules as well as robotic set up (17.1%) and technical skills (17.1%) such as knot tying.

## Discussion

This study showed the development and implementation of the IMRA Foundations in Robotic Surgery course utilising the Kern’s 6-step model for curriculum development.

**Fig. 3:**
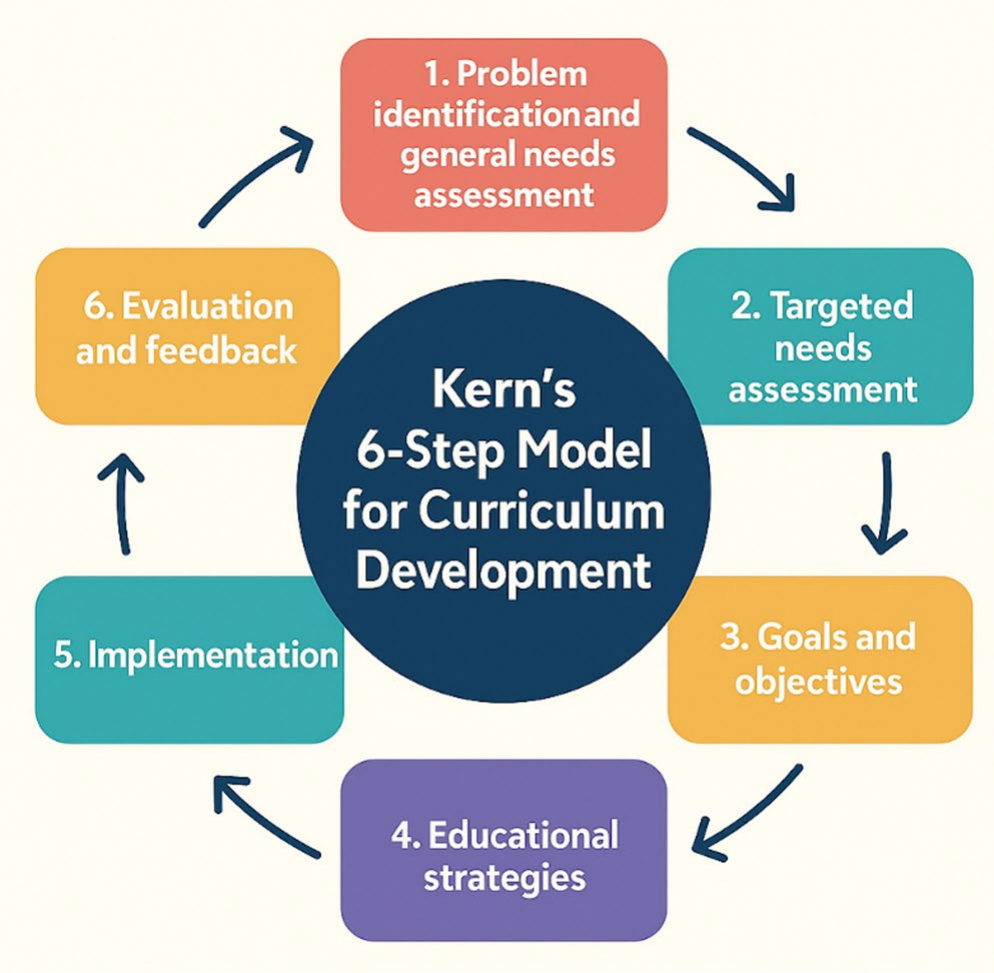
Kern’s six-step model for curriculum development

Kern’s 6-step model provided the scaffolding for the 11 self-directed online modules. Step 6, evaluation and feedback provided important areas for refinement and the course underwent several reiterations (Fig. 3).

Credentialing for robotic surgery remains heterogeneous as requirements differ markedly between institutions, as no standardised curriculum or mandated training modules currently exist. At present, there is no minimum training requirement before a trainee can sit at the console and attempt operative tasks. Traditionally, proctoring and informal mentoring have formed the backbone of robotic surgery training; however, with the IMRA modules, we aim to move toward greater consistency and standardisation.

Online learning has the potential to provide accessible, flexible, and efficient training for robotic surgery. The curriculum was designed for adult learners, using multimedia to strengthen concepts. The role of online learning in robotic surgery education has been evolving with the rapid advancement of technology and the increasing adoption of robotic surgery in various surgical specialties. Despite 98.9% of obstetric and gynaecology residents reporting that they had access to a robot, 35.0% reported having no robotic training, citing lack of personal time and lack of access to consoles as the biggest barriers. Moreover, 30% of respondents did not need to complete a robotics training course prior to working on the console, and 50% did not have formal evaluations during their robotic training [36]. 

Several studies have shown that online robotic surgery education can be effective in improving knowledge, skills, and confidence in performing robotic-assisted surgeries [7, 8]. A meta-analysis by Cook et al. (2008) found that internet-based learning, was associated with positive learning outcomes [7].

Mao et al. explored the use of videos for the online teaching of surgical skills. Their systematic review and meta-analysis found no significant difference between conventional and video-based education in teaching basic surgical skills. There was no statistically significant difference in skill proficiency between the two groups, (standardized mean difference of -0.02 (95% CI: -0.34, 0.30); *p* = 0.90). Moreover, three included studies found video learning to be superior to traditional teaching with the added benefits of flexibility [37]. 

Pape-Koehler et al. (2013) multimedia-based training on internet platforms can improve surgical performance. The study was a randomized controlled trial and involved expert robotic surgeons. The results showed that the group that received the multimedia-based training had significantly better surgical performance (mean score of 23.1/30 points) compared to the control group( mean score of 18.3/30 points) [38]. Our curriculum employs multimedia to provide a comprehensive understanding of robotic surgery, including technical and non-technical skills, that are often overlooked in other courses. Unlike Pape-Koehler et al., our curriculum is intended to be completed prior to hands-on training.

Decentralized training, has been shown to be an acceptable, effective and valid method of improving theoretical knowledge [39]. and providing accessibility and flexibility for trainees to learn at their own pace [40]. Moreover, online learning allows for a globalization and standardized adoption and training. In training for a population that is geographically dispersed, utilisation of video based learning is important to maximise efficiency during training events. Online education also offers accessibility to trainees in areas that may not have access to expensive training consoles, and allows for globalization and standardized training. Although online training cannot replace hands-on experience, the curriculum offers flexibility and overcomes barriers determined by irregular work schedules and locality.

Surgical teaching is usually delivered on the job in a mentorship-style, leading to huge variability in technical skills and exposure. We acknowledge that our small sample size may not reflect the needs of all surgical trainees. However, our curriculum provides a standardized baseline to ensure all trainees receive the same, high quality, up-to-date information. With clear and deliberate online training in robotic surgery significant reductions in the learning curve for novice surgeons can be achieved [10]. 

### Limitations

Knowledge acquisition was not assessed in this pilot study as the focus was on feasibility.

Now that the curriculum has been implemented internationally, future studies will incorporate objective measures of knowledge. We acknowledge this omission as a study limitation.

## Conclusion

It is imperative that standardized, reliable, and reproducible training modules are created for the safety and benefit of both doctors and patients. The Foundations in Robotic surgery online curriculum improves surgeon knowledge of robotic surgery and can improve access to initial robotic training.

## Appendix A

Literature review search strategy and search terms.

**Table:** 

Online Learning Resources in Robotic Surgery	
AND	E-learning Curriculum
AND	E-learning Modules
OR	Online Learning
OR	Remote Learning
OR	Video Learning

## Supplementary Information

Below is the link to the electronic supplementary material.

Supplementary Material 1

## Data Availability

Data is provided within the manuscript or supplementary information files.

## References

[1] Costello A, Challacombe B, Krishan S, McKenzie N, Monson J, Peters C et al (2023) Principles and Practice of Robotic Surgery, 1st Edition. 1st ed. Costello A, editor. Vol. 1. Elselvier

[2] Emergency Care Research Institute, Health Devices (2014) Top 10 Health Technology Hazards for 2015 a Report from Health Devices [Internet]. ECRI. Available from: https://www.ecri.org/Resources/Whitepapers_and_reports/Top_Ten_Technology_Hazards_2015.pdf

[3] Khan MTA, Patnaik R, Lee CS, Willson CM, Demario VK, Krell RW, Laverty RB. Systematic review of academic robotic surgery curricula. J Robot Surg. 2023 Jun;17(3):719-743. doi: 10.1007/s11701-022-01500-y. Epub 2022 Nov 21. PMID: 36413255. 10.1007/s11701-022-01500-y 36413255

[4] Lee JY, Mucksavage P, Sundaram CP, McDougall EM (2011) Best Practices for Robotic Surgery Training and Credentialing. Journal of Urology [Internet]. ;185(4):1191–7. Available from: https://robotictraining.org/wp-content/uploads/2013/07/RTN_for%20URO_GS_McDougallCredentialing.pdf 10.1016/j.juro.2010.11.067 21334030

[5] Zorn KC, Gautam G, Shalhav AL, Clayman RV, Ahlering TE, Albala DM et al (2009) Training, credentialing, proctoring and medicolegal risks of robotic urological surgery: recommendations of the society of urologic robotic surgeons. The Journal of urology [Internet]. [cited 2019 Oct 24];182(3):1126–32. Available from: https://www.ncbi.nlm.nih.gov/pubmed/19625032 10.1016/j.juro.2009.05.042 19625032

[6] Choules AP (2007) The use of elearning in medical education: a review of the current situation. Postgrad Med J 83(978):212–216 17403945 10.1136/pgmj.2006.054189 PMC2600032

[7] Cook DA, Levinson AJ, Garside S, Dupras DM, Erwin PJ, Montori VM (2008) Internet-Based learning in the health professions. JAMA 300(10):1181 18780847 10.1001/jama.300.10.1181

[8] Ruiz JG, Mintzer MJ, Leipzig RM (2006) The Impact of E-Learning in Medical Education. Academic Medicine [Internet]. ;81(3):207–12. Available from: http://workspace.unpan.org/sites/Internet/Documents/UNPAN93464.pdf 10.1097/00001888-200603000-00002 16501260

[9] Wittich CM, Agrawal A, Cook DA, Halvorsen AJ, Mandrekar JN, Chaudhry S, Dupras DM, Oxentenko AS, Beckman TJ. E-learning in graduate medical education: survey of residency program directors. BMC Med Educ. 2017 Jul 11;17(1):114. doi: 10.1186/s12909-017-0953-9. PMID: 28697744; PMCID: PMC5504987. 10.1186/s12909-017-0953-9 PMC5504987 28697744

[10] Chen R, Rodrigues Armijo P, Krause C, Siu KC, Oleynikov D (2019) A comprehensive review of robotic surgery curriculum and training for residents, fellows, and postgraduate surgical education. Surg Endosc 34(1):361–367 30953199 10.1007/s00464-019-06775-1

[11] Larvin M (2009) E-Learning in surgical education and training. ANZ J Surg 79(3):133–137 19317777 10.1111/j.1445-2197.2008.04828.x

[12] Kerfoot BP, DeWolf WC, Masser BA, Church PA, Federman DD (2007) Spaced education improves the retention of clinical knowledge by medical students: a randomised controlled trial. Medical Education [Internet]. ;41(1):23–31. Available from: http://onlinelibrary.wiley.com/doi/10.1111/j.1365-2929.2006.02644.x/abstract 10.1111/j.1365-2929.2006.02644.x 17209889

[13] Cook DA, Garside S, Levinson AJ, Dupras DM, Montori VM (2010) What do we mean by web-based learning? A systematic review of the variability of interventions. Med Educ 44(8):765–774 20633216 10.1111/j.1365-2923.2010.03723.x

[14] Thomas PA, Kern DE, Hughes MT, Chen BY (2015) Curriculum development for medical education: A six-step approach [Internet]. pure.johnshopkins.edu. Johns Hopkins University Press; Available from: https://pure.johnshopkins.edu/en/publications/curriculum-development-for-medical-education-a-six-step-approach

[15] Khamis NN, Satava RM, Alnassar SA, Kern DE (2015) A Stepwise model for simulation-based curriculum development for clinical skills, a modification of the six-step approach. Surg Endosc 30(1):279–287 25899812 10.1007/s00464-015-4206-x

[16] Tricco AC, Lillie E, Zarin W, O’Brien KK, Colquhoun H, Levac D et al (2018) PRISMA extension for scoping reviews (PRISMA-ScR): checklist and explanation. Ann Intern Med 169(7):467–473 30178033 10.7326/M18-0850

[17] Volkan Özben, Baca B (2019) Robotic-Assisted minimally invasive surgery. Springer eBooks. Springer Nature

[18] Kroh M, Sricharan C (2015) Essentials of robotic Surgery. Springer eBooks. Springer Nature

[19] Yuman Fong, Yanghee Woo, Woo Jin Hyung, Lau, C., Strong, V.E. and Springerlink (Online Service (2018). The SAGES Atlas of Robotic Surgery. 1st ed. Cham: Springer International Publishing, pp.4–530.

[20] Fundamentals of Robotic Surgery (FRS) [Internet]. Institute for Surgical Excellence (2022) [cited 2024 Mar 26]. Available from: https://www.surgicalexcellence.org/fundamentals-of-robotic-surgery-frs

[21] Smith R, Patel V, Satava R (2013) Fundamentals of robotic surgery: a course of basic robotic surgery skills based upon a 14-society consensus template of outcomes measures and curriculum development. Int J Med Rob Comput Assist Surg 10(3):379–384 10.1002/rcs.1559 24277315

[22] Satava RM, Stefanidis D, Levy JS, Smith R, Martin JR, Monfared S et al (2020) Proving the Effectiveness of the Fundamentals of Robotic Surgery (FRS) Skills Curriculum. Annals of Surgery 272(2):384–92. Available from: https://journals.lww.com/annalsofsurgery/Abstract/2020/08000/Proving_the_Effectiveness_of_the_Fundamentals_of.73.aspx 10.1097/SLA.0000000000003220 32675553

[23] Society of Robotic Surgery (2025) | SRS [Internet] Society of Robotic Surgery | SRS. Available from: https://srobotics.org/

[24] Da Vinci Education An integrated approach to professional education and programme services [Internet]. Intuitive.com (2023) Available from: https://www.intuitive.com/en-gb/products-and-services/da-vinci/education

[25] CMR Surgical(2025) [Internet] CMR Surgical. Available from: https://cmrsurgical.com/versius/training

[26] Medtronic Academy [Internet]. www.medtronicacademy.com. [cited 2024 Mar 26]. Available from: https://www.medtronicacademy.com/en-xp/home

[27] SIMULATION BASED TRAINING CURRICULUM | i Fundamentals of Robotic Gynecologic Surgery (FRGS) Curriculum [Internet]. [cited 2024 Mar 26]. Available from: https://simbionix.com/wp-content/pdf/curricula/RobotiX/Fundamentals_of_Robotic_Gynecologic_Surgery.pdf

[28] Carpenter BT, Sundaram CP (2017) Training the next generation of surgeons in robotic surgery. Robotic Surgery: Res Reviews 4:39–44 10.2147/RSRR.S70552 PMC6193443 30697562

[29] Urologic Robotic Surgery Online Course (2017) | AUA University [Internet]. auau.auanet.org. [cited 2024 Mar 26]. Available from: https://auau.auanet.org/node/4975

[30] Levy JS, Gharagozloo F (2021) Development of the fundamentals of thoracic robotic surgery curriculum. J Thorac Disease 13(10):6116–6122 34795962 10.21037/jtd-2019-rts-02 PMC8575813

[31] E-BRUS theoretical course - Uroweb [Internet] Uroweb - European Association of Urology. [cited 2024 Mar 26]. Available from: https://uroweb.org/education-events/e-brus-theoretical-course

[32] BRITISH ASSOCIATION OF UROLOGICAL SURGEONS (BAUS) ROBOTIC SURGERY CURRICULUM -- GUIDELINES FOR TRAINING (2015) [Internet] Available from: https://www.baus.org.uk/_userfiles/pages/files/Publications/Robotic%20Surgery%20Curriculum.pdf

[33] s.r.o LS ENYGO Introduction to Robotic Surgery Course (2024) [Internet]. ESGO - European Society of Gynaecological Oncology. Available from: https://esgo.org/courses/enygo-introduction-to-robotic-surgery-course2024/

[34] Robotic Thoracic Surgery Lung, Foregut, Mediastinal Surgery, and More | STS [Internet]. www.sts.org. [cited 2024 Mar 26]. Available from: https://www.sts.org/online-learning/robotic-thoracic-surgery-lung-foregut-mediastinal-surgery-and-more

[35] Robotic Surgery Webinar Series | European Society of Coloproctology [Internet] www.escp.eu.com. [cited 2024 Mar 26]. Available from: https://www.escp.eu.com/education/european-school-of-coloproctology/robotic-surgery-webinars

[36] Smith AL, Schneider KM, Berens PD (2010) Survey of obstetrics and gynecology residents’ training and opinions on robotic surgery. J Robotic Surg 4(1):23–27 10.1007/s11701-010-0176-0 27638568

[37] Mao BP, Teichroeb ML, Lee T, Wong G, Pang T, Pleass H (2022) Is online Video-Based education an effective method to teach basic surgical skills to students and surgical trainees? A systematic review and Meta-analysis. J Surg Educ 79(6):1536–1545 35933308 10.1016/j.jsurg.2022.07.016 PMC9356715

[38] Pape-Koehler C, Immenroth M, Sauerland S, Lefering R, Lindlohr C, Toaspern J et al (2013) Multimedia-based training on internet platforms improves surgical performance: a randomized controlled trial. Surg Endosc 27(5):1737–1747 23475016 10.1007/s00464-012-2672-y PMC3624003

[39] Lawrence C (2022) The role of the robotics coordinator: improving efficiency in a robotic surgery program. AORN J 115(3):239–249 35213048 10.1002/aorn.13625

[40] Kimmel HS, Carpinelli JD, Spak GT, Rockland RH (2020) A methodology for retaining student learning during the pandemic [Internet]. researchwith.njit.edu. ISTES; [cited 2024 Mar 26]. pp. 1–18. Available from: https://researchwith.njit.edu/en/publications/a-methodology-for-retaining-student-learning-during-the-pandemic

