# Supplementary material for: Development and implementation of the IMRA multiplatform Foundations in Robotic Surgery online learning curriculum
Source: J Robot Surg. 2026 Feb 26;20(1):298. doi: 10.1007/s11701-025-03091-w (PMC12935718; doi:10.1007/s11701-025-03091-w)
Supplement: Supplementary file 1 — Supplementary Material 1 [file 11701_2025_3091_MOESM1_ESM.docx]

Supplementary Data:

Online courses that were not specialty-specific that were identified were the following:

**The Fundamentals of Robotic Surgery**^20^ is a training program that offers four modules:

1)Introduction to surgical robotic systems

2) Didactic instructions to surgical robotic systems

3) Psychomotor skills curriculum

4) Team training and communication skills.

These modules aim to provide comprehensive training on the use of surgical robotic systems, covering both technical and communication skills necessary for successful surgeries. It focuses on 25 key metrics derived by consensus.^21^ The group divides skills into three key categories; team training, cognitive skills, and psychomotor skills.

The program adopts a WHO-like checklist approach and is currently being developed into a publicly accessible online education system. The model shows effectiveness, and is an encouraging step towards standardisation.^22^

**Society of Robotic Surgery**: The Society of Robotic Surgery provides online learning resources, including webinars, videos, and courses, for surgeons and healthcare professionals interested in robotic surgery. These resources span multiple surgical fields, including urology, gynaecology, colorectal, and general surgery.^23^

Device-specific, industry driven courses;

**The Da Vinci technology**^24^ training pathway offers two phases of training to those using the Da Vinci surgical system. Phase 1 covers the introduction to Da Vinci Surgery, while Phase 2 covers Da Vinci technology training in detail. The Intuitive course provided a series of rudimentary videos made by the robot manufacturer showing the different features and buttons on the Da Vinci robot and then a simple survey at the end of the course asking the student to name the various buttons and robot actions. No information about safety, communication, surgical techniques, or information from robotic surgeons was provided. It is worth noting, the most common didactic resource employed are the online modules developed by Intuitive Surgical, Inc.^3^

**The Versius training pathway** provides an e-learning online module that covers all aspects of the Versius robotic surgical system. This module covers the basic principles and advanced techniques of using the Versius system.^25^

**Medtronic** provides online educational materials for robotic surgery via the Medtronic Academy. These materials feature webinars, videos, and interactive sessions that are tailored to the HugoRAS system. ^26^

**Several specialty driven online courses were identified;**

**The Fundamentals of Robotic Surgery Curricula**^20^ have created two specialty specific modules:

1)The "Fundamentals of Robotic Gynaecologic Surgery (FRGS)" curriculum targets gynaecological robotic techniques for hysterectomies.^27^

2) "Fundamentals of Thoracic Robotic Surgery (FTRS)" is focused on the lobectomy procedure in thoracic surgery, covering essential anatomy and decision-making aspects.^28^

**The American Urologic Association** offers a Robotic Surgery Online Course that is specifically tailored for urologists.^29^ This course consists of nine modules covering the fundamentals, basics, and advanced urologic procedures using robotic surgery. The AUA requires a post- test score of 80%. Interestingly, the AUA pre-requisite is the Intuitive Surgical Online Training System, which has not yet been validated.^30^

**The European School of Urology** as part of the European Association of Urology offers the E-BRUS course, This course was specific to urology and approximately 60 minutes in duration. This course focused on general non-robotic laparoscopic skills and port placement, prostatectomy port placement, paediatric robotic surgery, upper tract reconstructive surgery and cystectomy. This course did not include information about the basic principles of robotic surgery.^31^The British association of Urological Surgeons (BAUS) recommend the intuitive e-learning be completed prior to completion of E-BRUS.^32^

## **European Network of Young Gynae Oncologists** provide an introduction to Robotic Surgery online module prior to a hands-on curriculum. The course is designed for young gynaecology surgeons with no prior robotic surgery experience.^33^

**The Society of Thoracic Surgeons** (STS) has developed the "Robotic Thoracic Surgery Comprehensive Training Program," which provides a detailed look at robotic thoracic surgery through seven on-demand sessions. These sessions include lectures, video demonstrations, and webinars led by faculty experts. The program is intended for thoracic surgeons, anaesthesiologists, nurses, and healthcare administrators who are interested in either initiating or improving their skills in robotic thoracic surgery.^34^

**The European School of Coloproctology** in collaboration with Intuitive has offered an extensive webinar series called "From basic skills to fine art" focused on colorectal robotic surgery. Comprising of four parts, the series delves into topics from fundamental principles of robotic colorectal surgery to advanced techniques for conditions like left flexure tumours and inflammatory bowel disease, as well as the importance of non-technical skills.^36^

The review highlighted a paucity of online learning resources for robotic surgery with most online resources being vendor-led. The majority of papers focused on an amalgamation of simulation-based training, online courses, and virtual reality platforms. Other courses were speciality specific.

**Content:** Robotic surgery education online resources typically cover a wide range of topics including the principles of robotic surgery, instrumentation and equipment, patient selection, as well as more specific topics such as surgical techniques, complications, and postoperative care.
